# Sports Organizations as Complex Systems: Using Cognitive Work Analysis to Identify the Factors Influencing Performance in an Elite Netball Organization

**DOI:** 10.3389/fspor.2019.00056

**Published:** 2019-11-04

**Authors:** Adam Hulme, Scott McLean, Gemma J. M. Read, Clare Dallat, Anthony Bedford, Paul M. Salmon

**Affiliations:** ^1^Centre for Human Factors and Sociotechnical Systems, University of the Sunshine Coast, Sippy Downs, QLD, Australia; ^2^School of Health and Sports Sciences, University of the Sunshine Coast, Sippy Downs, QLD, Australia

**Keywords:** cognitive work analysis, work domain analysis, complex systems, sport, netball

## Abstract

There is increasing interest in the adoption of a complex systems thinking approach when attempting to understand and optimize sports performance. Despite this, few studies have attempted to model elite sports organizations. The aim of this study was to use methods from the Cognitive Work Analysis (CWA) framework to develop a model of an elite netball organization and identify wider organizational functions and constraints influencing performance. Two phases of CWA were used: (i) Work Domain Analysis (WDA); and, (ii) Social Organization and Co-operation Analysis (SOCA). A WDA model of the elite netball organization was developed via subject matter expert (SME) workshops, and a SOCA was undertaken to identify the different functions, roles, and responsibilities of key actors within the organization. The findings identify various factors that influence performance. Functions that appear to provide a competitive edge are discussed, including a strong club ethos, a shared responsibility for performance, and a focus on player and staff health and well-being. Factors that potentially have a negative impact on performance include organizational priorities not related to playing netball, and additional coach and athlete roles beyond coaching, training, and playing. The implications for understanding and optimizing elite sports organizations are discussed.

## Introduction

The adoption of a complex systems thinking approach when attempting to understand elite sport performance is currently receiving traction in various sporting contexts (Cruickshank et al., 2015; Sadjad and Mitchell, 2016; Clacy et al., 2017; McLean et al., 2017; Mooney et al., 2017; Hulme et al., 2019a). In particular, it is recognized that sports organizations are characteristic of “complex sociotechnical systems,” and that a range of organizational factors interact to influence athlete performance (Fletcher and Wagstaff, 2009; Salmon, 2017; Rumbold et al., 2018; Hulme et al., 2019b). It has been argued that there is greater value in studying sports systems as a “whole,” rather than evaluating the relative contribution of their constituent “parts” in isolation (Hulme and Finch, 2015; Kleiner et al., 2015). Accordingly, systems analysis methods have been applied to examine sports organizations and systems in areas such as football (McLean et al., 2017), rugby (Clacy et al., 2017), and distance running (Hulme et al., 2017, 2019a). Given the reported utility of such applications, further systems thinking methods and approaches in other sports contexts have been encouraged (Hulme et al., 2019b).

Professional netball is gaining international popularity. In Australia, a newly formed professional netball league with arguably one of the highest playing standards in the world, the Suncorp Super Netball league, was established in February 2017. Whilst the popularity of netball and associated research applications are increasing (Croft et al., 2017; Bruce et al., 2018), complex systems thinking approaches have not yet been applied to the organizational level in elite netball. Compared to other fast ball invasion sports such as football, rugby, and basketball, there has been little research focusing on the range of factors external to the athletes and coaches that influence sports performance. The aim of this study was to use a well-known method from the discipline of human factors and ergonomics to provide an in-depth analysis of an elite Australian netball organization from a systems thinking perspective. This involved the development of a Work Domain Analysis (WDA) (Naikar, 2005, 2013) model of the netball organization, including an analysis of stakeholder functions, roles and responsibilities using the Social Organization and Co-operation Analysis (SOCA) component. The aim of the broader research program in which this work was undertaken was to identify new opportunities for optimizing system design with a view to further enhance organizational management and ultimately sports performance.

### Broader Research Program and Context

The present authors have recently published a WDA model of the “netball match system” to identify novel performance analysis measures that have not traditionally been considered in the sports science literature (McLean et al., 2019). Given the value and utility of this initial match focused analysis, the application of WDA and SOCA to the organizational context will not only demonstrate what the composition of a successful elite sports club looks like (i.e., from the perspective of task description, personnel allocation), but it might also expose areas in the system that require ongoing monitoring to ensure that desired performances are maintained over time. Before presenting the methods, there is a need to discuss what is known about the determinants of organizational performance in the wider sports management literature.

### What Is Known About Organizational Performance in Sport?

There is a large body of scholarly work that has studied the factors impacting the performance of national sports organizations and governing bodies. A useful starting point to frame the discussion can be found in a review that has synthesized the organizational performance management and measurement literature examining non-profit sports organizations (NPSOs) (O'Boyle and Hassan, 2014). In particular, there appears to be a scarcity of case studies focusing on the performance of local, profit-driven sports clubs, most likely due to the unique profile of such entities. Despite this lack of research, it can be argued that the determinants of performance associated with national sports organizations can offer useful insights that allow independent sports clubs to better evaluate and reflect on their own operations.

In summarizing, the NPSO performance management literature, the following four key messages can be extracted (O'Boyle and Hassan, 2014). First, a multi-dimensional approach to measuring and assessing NPSO performance is required, especially when considering that different stakeholders and members of an organization will have their own personal objectives to fulfill (Chelladurai and Haggerty, 1991; Bayle and Madella, 2002; Papadimitriou, 2002; Shilbury and Moore, 2006; Bayle and Robinson, 2007; Winand et al., 2010; Winand, 2011). For instance, the quality of the relations between an NPSO and the commercial sector (i.e., a predictor of economic stability) is of primary interest to governance teams and executives whereas the level of sports science support has been found to be an effective measure of performance in the eyes of technical staff (e.g., coaches, athletes) (Papadimitriou, 2002). This is one of the reasons why the concept of organizational performance cannot be reduced down to a single measure or factor (Herman and Renz, 1999; O'Boyle and Hassan, 2014). From a “whole systems” point of view, all performance-based measures are deemed to be of equal value and importance (O'Boyle and Hassan, 2014).

Second, the literature supports a long list of frequently recurring determinants of NPSO performance. Factors include human resource management and a functional volunteering structure, an interest in the health and well-being of athletes, efficient internal procedures, long-term planning and a distant outlook, ongoing customer communications, a positive outside image and perceived legitimacy, and the requisition and appropriate allocation of resources (Papadimitriou, 2002; Madella et al., 2005; Shilbury and Moore, 2006; Bayle and Robinson, 2007; Winand, 2011; Winand et al., 2013). Due to the complexity of NPSOs, organizational performance can only be understood when the interactions among identified determinants are studied in combination, rather than separately and alone (Winand et al., 2013). Indeed, reducing down organizational complexity to study the relative contribution of a single determinant cannot address the extent to which factors might compete for resources, or work synergistically in pursuit of a common system objective.

A third finding is the influence of stakeholder satisfaction on the future performance of NPSOs (O'Boyle and Hassan, 2014). Specifically, the level of satisfaction reported by stakeholders is a major determinant of whether NPSOs will meet their longer-term objectives (Papadimitriou and Taylor, 2000; Bayle and Madella, 2002; Madella et al., 2005; Shilbury and Moore, 2006; Bayle and Robinson, 2007; Winand et al., 2010). Similar to the concept of organizational performance, no clear definition of stakeholder satisfaction can be provided due to the various expectations and outcomes that different sponsors, shareholders, and investors will typically demand from an NPSO. Nevertheless, sports organizations should value and suitably reward those stakeholders who invest adequate resources and provide continued financial support over time, especially when there are multiple interested parties (O'Boyle and Hassan, 2014).

A final consideration relates to recommendations and directions for future sports management research. There is a need for new theoretical and methodological contributions that provide a holistic view of the various dimensions impacting organizational performance and management (O'Boyle and Hassan, 2014). The current study offers a novel contribution to the literature by applying a complex systems approach (i.e., WDA) to model the relationships among the objects, functions, values, and priorities of an elite sports club. Moreover, the SOCA component responds to the question of stakeholder satisfaction as this part of the analysis identifies which values and priorities are important to whom and relates this back to the functional purposes of the system. Similarly, the WDA model can be used to identify the extent to which the determinants of organizational performance appear in the netball system, including a description of the relationships between them and the actors who perform various role-related functions.

## Methods

This study applied two methods from the Cognitive Work Analysis (CWA) framework to produce a “complex systems model” of an elite netball organization. The methods include a WDA model of the netball club, followed by SOCA to identify key stakeholder functions, roles and responsibilities. From an ethical standpoint and for reasons of confidentiality, the netball club, the external stakeholder, and the specific roles reported by participants cannot be revealed. All data and analyses are presented in a non-identifiable manner. Ethical approval was granted by the institutional research ethics committee (project number A/17/1043).

### Overview of Cognitive Work Analysis (CWA)

Cognitive Work Analysis is a structured framework for analyzing and optimizing complex sociotechnical systems (Rasmussen et al., 1994; Vincente, 1999; Naikar et al., 2006; Jenkins et al., 2009; Stanton et al., 2018). There are five separate analytical phases within CWA, of which different combinations are typically used depending on the research aims. In the present study, two of those five phases were applied (Table 1).

**Table 1:** The two CWA phases, outputs, and elite-level netball organization examples.

**CWA phase**	**Outputs**	**Netball organization example**
WDA	Abstraction hierarchy of the sociotechnical system including the functional purpose, values and priority measures, purpose-related functions, object-related processes, and physical objects	Abstraction hierarchy describing the overall functional purposes (e.g., win the National Championship), values and priorities of the organization (e.g., uphold a positive reputation), the purpose-related functions (e.g., engage with the fanbase and community, play netball), object-related processes (e.g., branding and advertising), and physical objects and resources which afford those processes (e.g., office headquarters, information technology)
SOCA-WDA	Contextual overlay on the abstraction hierarchy to indicate the allocation of current functions across a range of identified actors	Abstraction hierarchy demonstrating which of the actors are currently performing the different functions required (e.g., which of the actors within the netball organization perform the function “financial management and accounting”)

*CWA, Cognitive Work Analysis; SOCA, Social Organization and Co-operation Analysis; WDA, Work Domain Analysis*.

### Work Domain Analysis

The first phase of CWA, WDA is used to provide an in-depth description of the system under analysis: in this case, the elite netball organization. The aim of WDA is to describe the system according to various levels of abstraction and decomposition, including its purposes and functions, as well as the various constraints imposed on the actions of any actor performing tasks within that system (Naikar, 2005, 2013).

The abstraction hierarchy method is used during WDA, and describes complex systems at the following five conceptual levels:

- Functional purpose: the overall purposes of the system and the external constraints imposed on its operation;
- Values and priority measures: the criteria that organizations use for measuring progress toward the functional purposes;
- Purpose-related functions: the generalized functions of the system that are necessary for achieving the functional purposes;
- Object-related processes: the functional capabilities and limitations of the physical objects within the system that enable the purpose-related functions; and,
- Physical objects: the physical objects and resources within the system that are used to undertake the generalized functions.

**Figure 1:**
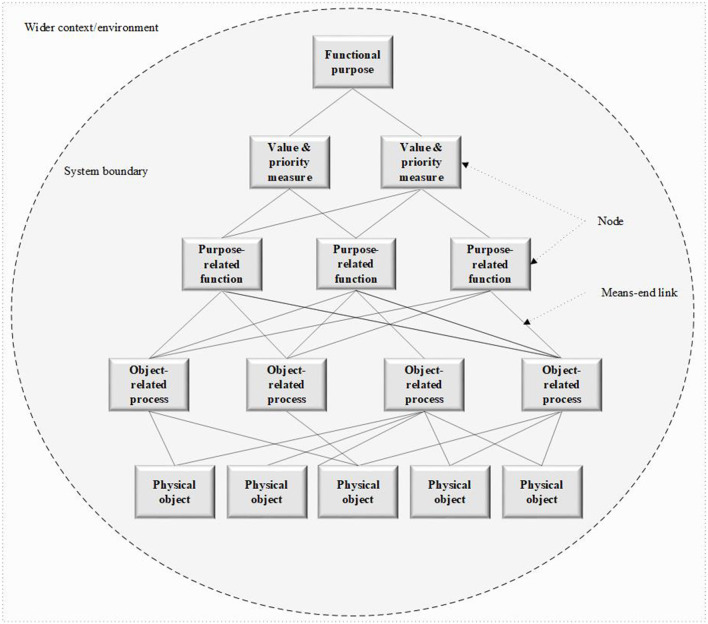
A generic WDA model template showing the relationships between the physical objects, object-related processes, purpose-related functions, values and priorities, and functional purposes of a system.

The WDA model uses “means-ends” links to link individual nodes across the five levels of abstraction described. For instance, any given purpose or function within a work system is generally achieved through several alternative or combined means originating from the level below. Not only does this illustrate the complexity of work systems, but it also shows that several functions at a particular level could compete for the same means (e.g., resources) that allow for their implementation. As a result, means-ends links exemplify the shared nature and tight coupling among the elements that comprise complex sociotechnical systems. A generic WDA model template, including means-ends links, is presented in Figure 1.

### Social Organization and Co-operation Analysis

The SOCA phase is used to identify how control tasks and functions are distributed amongst actors within the system, including how those different actors communicate and coordinate their actions (Jenkins et al., 2009; Stanton et al., 2018). The utility of SOCA is to understand how tasks are, and can be, optimally allocated, and how values and priorities, functions, and processes differ across different actors or organizations in a system. SOCA can be applied to the outputs from the first three phases of CWA (Jenkins et al., 2009), however, in the current study only the SOCA-WDA was undertaken (i.e., SOCA was overlaid on the WDA model output). The initial phase of SOCA involves identifying the current range of actors who “work” in the system (i.e., have designated responsibilities, perform tasks). From there, the analysis involves focusing on each of the WDA levels and identifying which of the actors currently contribute to (or use) the functional purposes, values and priorities, purpose-related functions, object-related processes, and physical objects.

### Materials and Methods

The study was undertaken by five researchers each with expertise in human factors, CWA, and sports systems analysis. Data were collected via a workshop with Subject Matter Experts (SMEs) from all levels of the elite netball organization (i.e., players through to executive members of the board).

### SME Characteristics and Expertise

A total of 13 SMEs (5 male, 8 female) aged between 21 and 66 years (*M* = 43.9 years, *SD* = 14.3 years) participated in the workshop. The total number of years that participants had been involved in elite-level netball varied between 1 and 24 years (*M* = 5.6 years, *SD* = 7.0 years). In terms of the general roles reported, there were two executive members of the board of directors including a senior university representative, one secretariat of the events board governance team from local council, two employees from the commercial sector (e.g., media, marketing, communications), two athlete support personnel (i.e., player healthcare and well-being), four individuals from the coaching, performance, and management team, two athletes, and one organizational administrator. The years of experience that participants had been working in their current role ranged from 1 to 24 years (*M* = 7.5 years, *SD* = 7.3 years).

### Procedures for Developing WDA and SOCA

Naikar's (2013) nine-step methodology for completing WDA for work systems was applied. An overview of the steps and the activities undertaken is provided.

#### Step One: Establish Aims and Purpose

Three members of the research team (AH, SM, PS) held a preliminary meeting with the netball organization to discuss the aims and expectations of the study. The aims of the analysis were to develop a systems model of the netball organization, including a description of the constraints around its operation, and to identify the specific tasks and activities that can be ascribed to different actors within the system.

#### Step Two: Anticipate Project Constraints

Consultation with the netball organization highlighted that due to dedicated training and competitive routines, it could be challenging for multiple people to meet at the same time, especially when this involves different actors from across the system (i.e., players and coaches through to senior management). As a result, the WDA model was developed during the off-season and had to be finalized prior to the first official game of the new season.

#### Step Three: Define the System Boundary

The analytical boundary around the netball organization had to be broad enough to capture the system in detail yet narrow enough to remain manageable and useful for addressing the study aims and purpose. Accordingly, the system boundary encapsulated the netball organization in its entirety and was extended to include the interests of key stakeholders including the local council, the official governing body of netball in Australia, sponsors, the external stakeholder, supporters, and the University.

#### Step Four: Classify System Constraints

The specific nature of sociotechnical systems can vary considerably. Some work systems exist to serve the user, whereas in others, it is the user that serves the system. In the wider human factors and ergonomics literature, this distinction has been referred to as the “causal-intentional continuum” of work systems (Rasmussen et al., 1994). Causal-based systems governed by physical processes are serviced entirely by workers who must ensure that system operation is congruent with the designers' objectives. For example, in the case of nuclear power production, working environments are purposefully designed and highly structured leaving little to no room for occupational error or latitude for behavior. In contrast to causal-based work systems, there are systems that allow for greater levels of actor intentionality. These systems vary in terms of what workers can and cannot do, however, organizational policies and legislation help to safeguard against adverse incidents and potentially dangerous scenarios. Typical examples of these systems are found in the engineering and healthcare contexts. On the other hand, public service institutions such as universities and sports organizations, afford even greater levels of actor intentionality, and control is shared between the system and its workers. In light of this, it was necessary to approximate where on the causal-intentional continuum the netball organization was located, as this enabled insight into the types of constraints to be modeled. Based on what the research team had discussed with the netball organization, the WDA focused on constraints around organizational performance more generally. The analysis did not therefore focus on environmental risk, manufacturing processes, or hard physical and engineered constraints.

#### Step Five: Locate Data Sources

Identifying and utilizing different sources of information to support WDA is recommended (Naikar, 2005, 2013). This study used a participatory approach to develop the WDA model by obtaining a highly relevant sample of SMEs who could both be distributed across and represent all levels of the netball organization. In addition, company reports, site maps, and online resources were used by the research team to familiarize themselves with the scope and scale of the netball organization. Workplace walkthroughs and unstructured field observations of training and game situations were also used.

#### Step Six: Construct the WDA

The WDA model was developed systematically during the SME workshop. In the workshop, the SMEs were divided into two groups, both of whom were asked to focus on completing the relevant WDA level concurrently. A list of WDA prompts for developing abstraction hierarchies for work systems was used to assist the SMEs in this process (Naikar, 2005). The results from each group were combined and refined during further discussion led by a facilitator with extensive experience in applying all phases of CWA (PS). After a draft WDA model was developed, the SMEs generated a list of actors that represented the relevant people and organizations (i.e., within the pre-defined system boundary; section Step Three: Define the System Boundary) and used this to undertake the SOCA phase. Guiding the SOCA analysis were the following questions corresponding to each of the five WDA levels: (i) For which actor(s) is this a functional purpose? (ii) Which actor(s) currently track progress toward the value or priority measure? (iii) Which actor(s) currently perform the purpose-related function? (iv) Which actor(s) currently use the object-related process? and, (v) Which actor(s) currently use the physical object?

#### Step Seven: Refine the Analysis

Four authors (AH, SM, GR, PS) applied the means-ends links connecting the nodes across the draft WDA model using the CWA software tool (Jenkins et al., 2007). Organizational reports, online resources, notetaking during field observations, and audio recordings obtained during the SME workshop were used to develop the means-ends links. Existing WDA guidelines suggest that means-ends links represent a series of “how-what-why” relations (Naikar, 2005). For example, in moving downwards through the levels of WDA abstraction, we asked: how is that functional purpose achieved? (i.e., what is the purpose-related function?). And, how is that purpose-related function enabled? (i.e., what is the physical object or resource?). Conversely, in moving upwards through the levels of WDA abstraction, the question was: why is it important to perform that function? And, why is the functionality afforded by that physical object or resource necessary?

#### Steps Eight and Nine: Review and Validate the WDA

The completed WDA-SOCA model was presented to six of the 13 initial SMEs for review and verification. The questions directed at participants followed a two-stage format. The first question sought a confirmatory response (or lack thereof), for example, “Do you agree that the purpose-related function of risk management is appropriate to include at this level?” And, “Do you agree that the actor(s) performing the function of risk management have been accurately represented?” The second set of questions were aimed to solicit an open-ended response, for example, “Can you think of any other physical object or resource which has not been described?” In terms of reviewing the means-ends links, the SMEs focused on at least two nodes and considered the “how-what-why” prompts as described in the previous step. Following the incorporation of feedback obtained from the SMEs during this step, a full color large-scale hardcopy version of the WDA model was provided to the netball organization, and participants were once again invited to provide any further comments and/or directly annotate over the model to indicate the need for additional modifications.

## Results

**Figure 2:**
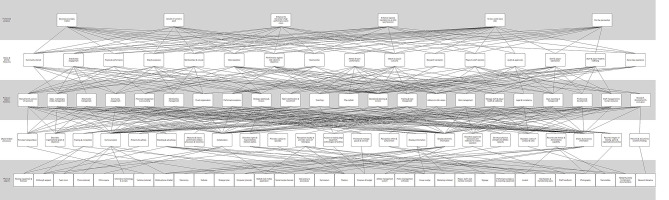
The WDA model of the netball organisation.

**Figure 3:**
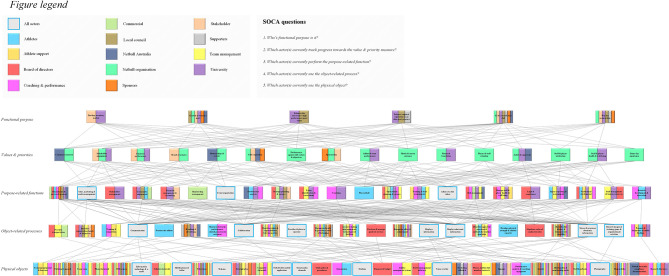
The WDA-SOCA model of the netball organisation.

The WDA and WDA-SOCA models of the netball organization are presented in Figures 2, 3, respectively. The colored shading in Figure 3 can be cross-referenced with the relevant SOCA questions as found on the “Figure legend.” The list of SOCA actors and their associated descriptions is presented in Table 2. Specific modifications to the WDA-SOCA model following step eight of the nine step methodology can be viewed in the Supplementary Material.

**Table 2:** The list of SOCA actors identified during the SME workshop as viewed in Figure 3.

**Actors**	**Description**
Athletes	Regular players selected to represent the netball club at competitive events
Athlete support	Provide healthcare, both physical and psychological, to the athletes directly
Board of directors	High-ranking decision makers including the Chief Executive Officer
Coaching and performance	Work directly with the athlete, including strength and conditioning specialists
Commercial	Promote the club through general media and marketing-related activities
Local council	Events board governance team concerned with regional sports and activity profiles
Netball Australia	The official governing body of netball in Australia
Netball organization	Excludes Netball Australia, sponsors, the stakeholder, supporters, and the university
Sponsors	Partners supporting the organization through the provision of products and services
Stakeholder	The external organization with an interest in the club's objectives and policies
Supporters	Enthusiasts and fans of the netball organization and its activities
Team management	Provides direction, instruction, and guidance to athletes and other staff members
University	Public institution with an interest in securing a future for the netball organization

### Functional Purposes

The functional purposes level highlights the reasons why the netball organization exists and indicates the primary objectives of the system. Six functional purposes were identified: (i) “develop secondary market;” (ii) “growth of women's sport;” (iii) “enhance the University's high-performance sport vision;” (iv) “enhance regional reputation as an elite sport precinct;” (v) “to be a world class club;” and, (vi) “win the premiership.” In terms of the SOCA, seven actors (i.e., the netball organization, the external stakeholder organization, the University, local council, supporters, sponsors, Netball Australia) were assigned to the functional purpose “to be a world class club.” Conversely, “develop secondary market” was of concern to the external stakeholder organization and the University only. The national netball governing body was assigned to two functional purposes, namely, “growth of women's sport” and “to be a world class club.” The purpose “win the premiership,” was perceived to be of interest to six actors (i.e., the netball organization, the external stakeholder, the University, local council, supporters, sponsors).

### Values and Priority Measures

The values and priority measures level of the WDA model contain criteria that are used to assess the netball organization's progress toward achieving its functional purposes. There were 16 values and priorities identified. In a broad sense, they can be grouped under the categories of community engagement, stakeholder management, organizational reputation, athletic performance, and player and staff health and well-being. According to SOCA, the netball organization is currently involved in tracking the progress of all 16 values and priority measures. The other actors to feature on this level were Netball Australia (i.e., “community interest,” “memberships & crowds,” “club reputation,” “audits & approvals”), the external stakeholder organization (i.e., “stakeholder engagement,” “financial performance,” “brand awareness,” “club reputation,” “sponsorship”), the University (i.e., “financial performance,” “athlete & team performance,” “research translation,” “audits & approvals,” “staff & player health & well-being”), and sponsors (i.e., “club reputation,” “sponsorship”). Supporters and local council do not currently track any of the values and priority measures.

### Purpose-Related Functions

The purpose-related functions level describes the broad activities that must be fulfilled in order for the system to achieve its overall functional purposes. The 22 functions at this level impose demands on the resources and object-related processes as found on the levels below. The SOCA shows that the University (20), the board of directors (15), coaches (13), and athletes (12) perform the most functions at this level (functions in parentheses). All actors are currently involved in performing the functions of “sales, marketing & media management,” “event organization,” and “adhere to club values.” Likewise, “recruitment & service of sponsors,” “strategic planning & reviews,” and “operational planning & reviews” were a further three generalized functions that are performed by a majority of actors. The tasks of “membership management” and “play netball” were ascribed to the “commercial” and “athlete” actors only.

### Object-Related Processes and Physical Objects

The lower two levels of the WDA model include the physical objects and resources contained within the netball organization, as well as the processes that those objects and resources afford. There were 32 physical objects and 23 object-related processes. The objects that are currently used by all actors include “information technology & e-mail,” “mobile phone & tablet,” “website,” “netball club mobile application,” “social media channels,” “stadium,” “venue overlay,” and “photography.” Certain objects were identified as having multiple means. For example, “clothing & apparel” is used by a range of different actors and enables varied processes such as “training & competition” and “branding & advertising.” Likewise, the “netball club mobile application” enables not only “collaboration,” but it also “recognizes loyalty & provides a sense of belonging.” Conversely, some objects such as “club policy & procedures” and “finances & budget” were ascribed to a single actor (i.e., “board of directors”).

## Discussion

The aim of this study was to develop a complex systems model of an elite netball organization and identify how specific tasks and functions are currently distributed among key actors within the system. The analysis represents the first time that methods from the CWA framework have been applied to describe the functional structure of an elite sports organization. The main findings are discussed below in terms of the insights regarding organizational management and the optimization of elite sports performance.

### Complexity of Elite Netball

The findings provide a clear demonstration of the complexity of the netball organization and emphasize that many factors outside of coaches and players can potentially influence player, team, and organizational performance (Hulme et al., 2019b). The WDA-SOCA model identified multiple purposes, values, functions, processes and objects, and showed the diverse set of actors who share the responsibility for sports management and organizational performance. In this sense, the netball team's performance is truly an emergent property of the broader netball organization, and it is the complex interactions that take place between many actors, objects, and technologies from across the system that determine whether a desirable outcome is achieved. In addition, the means-end links in the WDA model indicate a high level of interconnectivity among different processes and functions, suggesting that the effect of intervening or making a change to a single factor, decision, or action at one level will influence multiple components across the whole netball system. This finding is consistent with existing research that suggests organizational performance in sport can only be fully understood when the interactions among determinants are studied in combination and modeled holistically (Winand et al., 2013).

### The Ingredients of Success

Before examining aspects of the WDA that indicate areas for improvement, it is first worth looking at the structure of the organization to identify what works well (i.e., the team in question won the last two grand finals in both of its first two seasons). There is much that other elite sports organizations can learn from the analysis, especially given that the WDA model includes functions beyond those of merely training, coaching, and playing netball. First, the SOCA provides a clear indication of the extent to which there is a strong shared responsibility for performance within the organization. The majority of the purpose-related functions and object-related processes are undertaken by multiple actors, and there are few that are the responsibility of one actor alone. This shared responsibility ethos is a key feature that relates to the multi-dimensional nature of NPSO performance measurement more generally (Chelladurai and Haggerty, 1991; Bayle and Madella, 2002; Papadimitriou, 2002; Shilbury and Moore, 2006; Bayle and Robinson, 2007; Winand et al., 2010; Winand, 2011) and appears to be just as important at the elite sports club level. Second, the WDA model demonstrates the positive influence of having a strong high-performance sports culture (Henriksen, 2015; Maitland et al., 2015). Specifically, the purpose-related function “adhere to club values” is heavily linked to many of the values and priorities, demonstrating its importance in attaining the functional purposes. In addition, the node “adhere to club values” within the WDA model is supported by multiple processes that the organization performs to ensure that their strong club values and ethos are maintained. Third, the analysis shows a strong emphasis on managing staff and player health and well-being. This purpose-related function is linked to many nodes above it and has many nodes linked to it from the object-related processes level. Fourth, “research development and innovation” was identified as a purpose-related function that may not be performed by all elite netball organizations. Based on the club's strong connection with the University, the netball organization not only values the importance of research, it actively engages in it. Effective translation of this research likely provides an edge over competing organizations who are not affiliated with a university. Fifth and finally, the WDA-SOCA model of the netball organization appears to resemble many of the key determinants that are associated with high performing NPSOs (O'Boyle and Hassan, 2014).

### Compatibility of Functional Purposes

Six functional purposes of the netball organization were identified. Whilst some are to be expected from an elite sport organization, e.g., “win the premiership,” other influential functional purposes were identified, e.g., “growth of women's sport” (desired by the local council); “enhance the University's high-performance sport vision” (desired by the University); and “enhance regional reputation as an elite sport precinct” (desired by the local council). Such diversity in purposes not only contributes to the complexity of the system, but it also raises the question of whether the organization will be able to maintain optimal levels of performance whilst still pursuing its other functional purposes. It is therefore important to consider the extent to which the functional purposes are mutually supportive, as opposed to being conflicting and problematic as found in other CWA analyses (Salmon et al., 2016). Broadly speaking, the functional purposes in this case appear to be largely compatible in that success on-court will enable attainment of many of the other purposes. However, possible exceptions to this include the purposes of, “develop secondary market” and “win the premiership.” Specifically, there is a potential trade-off between having to expend resources to achieve performance-related goals, against the need develop and expand the secondary market. The notion of potentially conflicting functional purposes relates to the competing values framework, or “Quinn model,” which explains that organizational effectiveness is continually being “pulled” in different directions based on two main dimensions; flexibility/stability, and an internal/external focus (Quinn, 1988). Stated simply, organizations are required to be adaptable and dynamic, but also stable and controlled. Likewise, they have to be integrated and unified, but equally competitive and distinct from outside competition. The competing values framework can be used by organizations to diagnose possible conflict, whether related to resource expenditure against profitability, as is the case in the current analysis, or to identify where current or future sources of cultural, managerial, or individual role-related tensions might occur (Denison and Spreitzer, 1991; Shilbury and Moore, 2006).

Aside from the idea of conflicting or supportive functional purposes, it would be useful for the netball organization to reflect on whether its six purposes can be reasonably justified (i.e., are there too many reasons for its existence?), as well as *why* a particular functional purpose may be prioritized over the others. Although the SOCA component of the analysis can only indicate which of the functional purposes “belong” to the various different stakeholders and members, it could be hypothesized that each stakeholder may end up justifying the legitimacy of the entire system, including its multiple goals and purposes, given that they are motivated to think of the organization as fair, just, and desirable to be a part of Haack and Sieweke (2018). Indeed, system justification theory explains that different members of a social system (in this case the organization) tend to support the status quo, even if they are disadvantaged as a result of certain outcomes. Although Haack and Sieweke's (2018) thesis centers around the legitimization of social inequality by drawing on system justification and social judgment perspectives, there are nevertheless parallels to be drawn with the netball organization. A case in point are the athletes and coaches who are required to undertake various purpose-related functions beyond playing and training as a means of contributing toward the functional purpose of “enhance the University's high-performance sports vision.” The athletes and coaches in context of the wider netball organization could therefore be seen as both a benefactor (i.e., “win the premiership”) and victim (i.e., “enhance the University's high-performance sports vision,” “develop secondary market”) of the system's overarching functional purposes, despite the possibility of feeling a general sense of belonging and unity. On the other hand, the functional purposes may not necessarily be held in equal regard by the team, or all its stakeholders. If this is the case, then there is scope for the organization to symbolically acquiesce with some requirements (e.g., “growth of women's sport”), while prioritizing other goals (i.e., “win the premiership”). Unfortunately, the present analysis cannot provide such insight, and so an interesting line of further research should aim to investigate whether the theory of system justification in the sports organizational context could contribute new knowledge to the area. In sum, decision makers should remain cognizant of the main purposes for why the netball organization exists. Such knowledge could be used in strategic planning activities to ensure that system performance is maintained over time, and that its goals are, for the most part, congruent with the interests of all its members.

### Non-sport Related Functions and Athlete Workload

The findings show that a multitude of functions are required to support operation of the netball organization. Whilst this is not surprising, it is important to note that many of the functions do not relate to playing netball or optimizing athlete and team performance (e.g., coaching, training). Purpose-related functions relating to broader organizational working practices include, the “recruitment & service of sponsors,” “sales & marketing,” “stakeholder & community engagement,” “membership management,” “strategic & operational planning,” “talent identification,” “risk management,” and “staff & player professional development.” The functions relating directly to playing netball include, “training & competition,” “managing workloads,” and “coaching & performance analysis.” The SOCA-WDA model indicates that outside of these “playing” functions, the athletes and coaches are engaged in many other functions (12 and 13 out of a possible 22, respectively). For example, players are required to undertake functions such as “community engagement,” “operational planning,” “sales, marketing & media management,” and the “recruitment & service of sponsors.” A key consideration for optimizing performance is that athlete workload management not only relates to training and playing, but also covers non-playing functions such as those included in the WDA. Previous research makes the case that the health and well-being of athletes can, for better or worse, be traced directly back to a number of “organizational stressors” or factors that reside beyond the immediate intrapersonal and team level (Fletcher and Wagstaff, 2009). Accordingly, the overall stress experience in athletes, up to and including wider organizational influences, is a necessary consideration if match performances are to be optimized (Fletcher and Wagstaff, 2009). The extent to which the workload associated with these additional WDA functions may degrade coach and athlete performance in the elite netball context requires further investigation.

### Values and Priorities

Similar to the purpose-related functions level, the values and priorities level includes multiple nodes that are not directly related to playing the game of netball. This highlights a range of measures that sports organizations are required to assess when evaluating their own performance. As shown by the SOCA, it is the netball organization themselves who are primarily enacting the value and priority measures to track progress toward the functional purposes described. This is surprising given that various other actors are associated with different functional purposes at the level above. Given that the netball organization may not have access to all data relevant for tracking the necessary system priorities, new approaches to measuring organizational performance may be required. Specifically, the workload imposed on the netball organization could be problematic and so it is recommended that all stakeholders explore options to support the reallocation and/or redistribution of roles regarding measurement of progress toward the functional purposes. New mechanisms for collecting, sharing, and analyzing data could be introduced and trialed so that the netball organization can continue to measure important criteria, albeit with the support from other actors who are as equally interested in key outcomes. Establishing these new mechanisms at an early stage in the organization's development will come at an initial cost in terms of time and finances, however it might be a useful pre-emptive action prior to the manifestation of potential issues that typically parallel organizational growth (O'Gorman, 2001; Sinclair, 2007). It is recommended that the model presented be used to identify combined possibilities for new multi-stakeholder value and priority measures. Indeed, stakeholder satisfaction in the NPSO literature has been identified as a leading determinant of organizational performance (Papadimitriou and Taylor, 2000; Bayle and Madella, 2002; Madella et al., 2005; Shilbury and Moore, 2006; Bayle and Robinson, 2007; Winand et al., 2010), and it may be the case that the stakeholders associated with a specific functional purpose are given responsibility for tracking progress toward that purpose as a means of assigning a degree accountability and ownership.

### Contribution of CWA and Systems Analysis Methods and Models

On closer inspection of the sports management literature, including studies that cover topics aligned with performance measurement (O'Boyle and Hassan, 2014), institutional theory (Washington and Patterson, 2011), and organizational culture (Maitland et al., 2015), it appears that there is a paucity of research that has used systems thinking approaches and analysis methods. Whilst the studies reported within current reviews are underpinned by a wide range of theoretical models (e.g., resource dependency theory, competing values approach) (Madella et al., 2005; Shilbury and Moore, 2006) to support the application of both quantitative and qualitative study designs (e.g., ethnographic inquiry, thematic analyses), there is an opportunity to build on the capacity of existing research by modeling and visualizing the interactions among multiple levels of organizational systems in sport. The use of the two constituent CWA methods in this study, namely WDA and SOCA, could be applied in future applications to facilitate an understanding of the systemic processes and constraints impacting the effective operation of other sports organizations. This is because systems analysis methods can highlight areas of current or possible future organizational conflict, such as when a function or purpose is demanding of the same set of resources (e.g., Quinn, 1988; Denison and Spreitzer, 1991). Moreover, the CWA framework includes a total of five analytical phases (Vincente, 1999; Stanton et al., 2018), and so future studies are encouraged to explore what the other three methods, control task (activity) analysis, strategies analysis, and worker competencies analysis, could offer to the field of sports management research.

### Limitations and Future Research-Based Considerations

This study has notable limitations. The first limitation pertains to the sample size of SMEs who developed, refined, and validated the WDA model. Although an evenly distributed sample of experts was obtained from across the netball organization, it would have been beneficial to have included at least one member from the external stakeholder organization. Accordingly, a senior university member and executive of the board of directors served to represent the views of a suitable stakeholder representative. A second limitation relates to the descriptive and exploratory use of the CWA approach. Specifically, the measurable impact associated with the implementation of real-world performance enhancing strategies and interventions designed to optimize operations cannot be evaluated in this case. This is an avenue for further research and is dependent upon the uptake of the recommendations by decision makers and stakeholders.

## Conclusion

This study has shown that elite netball organizations are complex sociotechnical systems involving a range of actors who use a large number of physical objects and resources in order to carry out a diverse set of functions, many of which sit outside the expected functions of training and playing netball. Important conclusions from the analysis are that currently there may be competing functional purposes, that athletes and coaches may be over-tasked with non-sport related functions and processes, and that the responsibilities for measuring progress toward functional purposes could be revisited. It is concluded that methods found in the CWA framework, in this case WDA and SOCA, are useful approaches for understanding and describing sports organizations and the structural intricacies that surround and potentially influence elite sports performance. A great deal of work in the sports sciences has focused on the behaviors of athletes, coaches, and sports teams in relation to performance optimization. This study demonstrates that although traditional athlete and team-centered approaches are useful and should continue to be used, examining broader organizational structures (including the main system priorities) can provide insights for better optimizing sports performance at the individual-level. Future applications of systems analysis methods in the sports management field and in different sporting contexts are encouraged.

## Data Availability Statement

Qualitative data derived from participant workshops are presented in aggregate. Individual participant responses will not be made publicly available.

## Ethics Statement

The studies involving human participants were reviewed and approved by University of the Sunshine Coast Human Research Ethics Committee. The patients/participants provided their written informed consent to participate in this study.

## Author Contributions

All authors contributed to data collection, analysis and interpretation, writing, and review of the manuscript.

### Conflict of Interest

The authors declare that the research was conducted in the absence of any commercial or financial relationships that could be construed as a potential conflict of interest.
