# Supplementary material for: Sports Organizations as Complex Systems: Using Cognitive Work Analysis to Identify the Factors Influencing Performance in an Elite Netball Organization
Source: Front Sports Act Living. 2019 Nov 4;1:56. doi: 10.3389/fspor.2019.00056 (PMC7739835; doi:10.3389/fspor.2019.00056)
Supplement: Supplementary file 1 [file Table_1.DOCX]

**Supplementary Materiel**

**Table S1:** Specific changes that were made to means-ends links relationships following step eight (i.e. the review stage) of WDA model development. Four members of the research team (Hulme, Mclean, Read, Salmon) and six SMEs participated. Many changes involved the addition of new means-ends links (🡪) that had not been accounted for, or the removal of existing mean-ends links (🡨) that were thought to be incorrect.

| **Values & priority measures to functional purpose** |
| --- |
| ‘Athlete and team performance’ 🡪 ‘Growth of women’s sport’ |
| ‘Performance against club values & objectives’ 🡪 ‘Enhance the University’s high-performance sport vision’ |
| ‘Player & staff retention’ 🡪 ‘Enhance University’s high-performance sport vision’ |
| ‘Club reputation’ 🡪 ‘Enhance regional reputation as an elite sport precinct’ |
| ‘Sponsorship’ 🡪 ‘Enhance regional reputation as an elite sport precinct’ |
| ‘Club reputation’ 🡪 ‘To be a world class club’ |
| ‘Staff and player health & wellbeing’ 🡨 ‘To be a world class club’ |
| ‘Financial performance’ 🡪 ‘Win the premiership’ |
| **Purpose-related functions to values & priority measures** |
| ‘Play netball’ 🡪 ‘Stakeholder engagement’ |
| ‘Community engagement’ 🡪 ‘Stakeholder engagement’ |
| ‘Event organisation’ 🡪 ‘Membership & crows’ |
| ‘Talent identification & recruitment’ 🡪 ‘club reputation’ |
| ‘Research development & innovation’ 🡪 ‘Club reputation’ |
| ‘Performance analysis’ 🡪 ‘Performance against club values & objectives’ |
| ‘Manage staff & player health & well-being’ 🡪 ‘Performance against club values & objectives’ |
| ‘Community engagement’ 🡪 ‘Sponsorship’ |
| ‘Adhere to club values’ 🡪 ‘Sponsorship’ |
| ‘Manage staff & player health & well-being’ 🡪 ‘Athlete & team performance’ |
| ‘Performance analysis’ 🡪 ‘research translation’ |
| ‘Talent identification & recruitment’ 🡪 ‘Player & staff retention’ |
| ‘Staff management & human resources’ 🡪 ‘Audits & approvals’ |
| ‘Injury management’ 🡪 ‘Audits & approvals’ |
| ‘Play netball’ 🡪 ‘Staff & player satisfaction’ |
| ‘Training & load management’ 🡪 ‘Player satisfaction’ |
| ‘Coaching’ 🡪 ‘Staff & player health & well-being’ |
| ‘Play netball’ 🡪 ‘Staff & player health & well-being’ |
| ‘Event organisation’ 🡪 ‘Game day experience’ |
| ‘Financial management & accounting’ 🡪 ‘Performance against club values & objectives’ |
| **Object-related processes to purpose-related functions** |
| ‘Access to leading edge equipment, technologies & facilities’ 🡪 ‘Research development & innovation’ |
| ‘Stipulates codes of conduct & rules’ 🡪 ‘Manage staff & player health & well-being’ |
| ‘Displays electronic information’ 🡪 ‘Operation planning & reviews’ |
| **Physical objects to object-related processes** |
| ‘Performance analysis & coaching equipment’ 🡪 ‘Stores & processes electronic information’ |
| ‘Performance analysis & coaching equipment’ 🡪 ‘Displays electronic information’ |
| ‘Netball club mobile application’ 🡪 Recognises loyalty & provides a sense of belonging’ |
| ‘Awards’ 🡪 ‘Recognise loyalty & provides a sense of belonging’ |

**Table S2:** Specific changes that were made to SOCA following step eight (i.e. the review stage) of WDA model development. Four members of the research team (Hulme, Mclean, Read, Salmon) and six SMEs participated.

| **Functional purpose** |
| --- |
| University coded to ‘Develop secondary market’ |
| University coded to ‘Growth of women’s sport’ |
| Supporters coded to ‘Enhance regional reputation as an elite sport precinct’ |
| **Values & priority measures** |
| University coded to “Financial performance’ |
| University coded to ‘Athlete & team performance’ |
| University coded to ‘Audits & approvals’ |
| University coded to ‘Staff & player health & well-being’ |
| Netball Australia removed from ‘Game day experience’ |
| Local council removed from ‘Membership & crowds’ |
| Local council removed from ‘Community interest’ |
| **Purpose-related functions** |
| University coded to ‘Recruitment & service of sponsors’ |
| University coded to ‘Stakeholder management’ |
| University coded to ‘Community engagement’ |
| University coded to ‘Performance analysis’ |
| University coded to ‘Talent identification & recruitment’ |
| University coded to ‘Coaching’ |
| University coded to ‘Operational planning & reviews’ |
| University coded to ‘Training & load management’ |
| University coded to ‘Risk management’ |
| University coded to ‘Manage staff & player health & well-being’ |
| University coded to ‘Legal and compliance’ |
| University coded to ‘Injury reporting and management’ |
| University coded to ‘Professional development’ |
| University coded to ‘Staff management and human resources’ |
| Stakeholders removed from ‘Community engagement’ |
| Stakeholders removed from ‘Membership management’ |
| Commercial coded to ‘Operational planning & reviews’ |
| Netball Australia coded to ‘Performance analysis’ |
| Athletes coded to ‘Community engagement’ |
| Coaching & performance coded to ‘Research development & innovation’ |
| **Object-related processes** |
| All actors coded to ‘Communication’ |
| Local council coded to ‘Branding & advertising’ |
| **Physical Objects** |
| All actors coded to ‘Website’ |
| Local council coded to ‘Strategic plan’ |
| Stakeholders coded to ‘Signage’ |
| Board of directors coded to ‘Research literature’ |
| Team manager coded to ‘Fortis management software’ |

**Table S3:** Specific changes that were made following step eight (i.e. the review stage) of WDA model development based on a final review of the analysis supplied to the netball organisation in full colour hardcopy format.

| **Functional purpose (n/a)** |
| --- |
| **Values & priority measures** |
| Stakeholders coded to ‘Stakeholder engagement’ |
| Stakeholders coded to ‘Financial performance’ |
| Sponsors coded to ‘Club reputation’ |
| Stakeholders coded to ‘Club reputation’ |
| Netball Australia coded to ‘Club reputation’ |
| Sponsors coded to ‘Sponsorship’ |
| Stakeholders coded to ‘Sponsorship’ |
| **Purpose-related functions (n/a)** |
| **Physical objects (n/a)** |
